# Evolving Decongestion Strategies in the Management of Acute Heart Failure

**DOI:** 10.1007/s11936-025-01125-y

**Published:** 2025-10-18

**Authors:** Jeffrey George, W. H. Wilson Tang

**Affiliations:** 1 Case Western Reserve University, Cleveland, OH USA; 2 Department of Cardiovascular and Metabolic Sciences, Cleveland Clinic Research, Cleveland, OH USA; 3 Kaufman Center for Heart Failure Treatment and Recovery, Heart Vascular and Thoracic Institute, Cleveland Clinic, Cleveland, OH USA; 4 Cleveland Clinic Lerner College of Medicine of Case Western Reserve University, Cleveland, OH USA

**Keywords:** Acute heart failure, Diuretics, Guideline-directed medical therapy

## Abstract

**Purpose of Review:**

To provide an overview of the latest evidence-based treatment strategies for acute heart failure (HF), with a significant focus on recent clinical trials and their associated implications within clinical practice.

**Recent Findings:**

Recent studies have highlighted the effectiveness of urine sodium-guided decongestion, segmental nephron blockade, disease-modifying therapies, and the use of maintenance diuretics. These approaches have demonstrated significant improvements in patient outcomes, including better decongestion, reduced hospital admissions, and improved survival rates.

**Summary:**

The findings suggest a multifaceted, evidence-based approach is necessary for the effective management of acute HF. Implementing urine sodium-guided decongestion and segmental nephron blockade can enhance diuretic efficiency while disease-modifying therapies improve long-term outcomes. Maintenance diuretics play a crucial role in preventing recurrent hospitalizations.

## Opinion statement

Based on recent clinical trials, the optimal management strategy of acute heart failure (HF) involves a combination of urine sodium-guided decongestion with segmental nephron blockade and early initiation of disease-modifying therapies. Urine sodium-guided decongestion allows for precise adjustment of diuretic therapy with targeted goals for enhancing decongestion. Segmental nephron blockade leveraged different parts of the nephron to achieve a more effective decongestant approach. Disease-modifying therapies, including neurohormonal antagonists and SGLT2 inhibitors, should be initiated early to improve long-term outcomes.

## Introduction

Acute heart failure (HF, or “worsening HF” in some contexts) is characterized by the rapid onset or worsening of HF symptoms such as shortness of breath or fluid retention, typically requiring urgent medical interventions. Sometimes it is triggered by an inciting event that can lead to the inability of the heart to meet the body’s circulatory demands, such as myocardial infarction or arrhythmias [1, 2]. Acute HF can be life-threatening, with high rates of hospitalization and mortality, especially in patients who experience recurrent episodes [3]. Given the acute and severe nature of this condition, prompt recognition and treatment are critical for stabilizing the patient and preventing long-term complications.

In the setting of hospitalized patients with acute HF, the primary clinical challenge is not only acute stabilization and relief of congestion but also restoring euvolemia to facilitate initiation or uptitration of long-term disease-modifying therapies. The rationale is clear – signs and symptoms of volume overload, such as dyspnea, fatigue, orthopnea, and edema, are the cardinal symptoms that prompt patients to seek hospital admissions, and decongestion with diuretics is the primary treatment goal for acute symptom relief. While inpatient guideline-directed medical therapy (GDMT) initiation and uptitration has growing justification, persistent volume overload and inadequate decongestion can impede drug tolerance and potentiate adverse effects such as hypotension, kidney dysfunction, or other unwanted consequences if patients are not adequately stabilized and decongested.

**Fig. 1:**
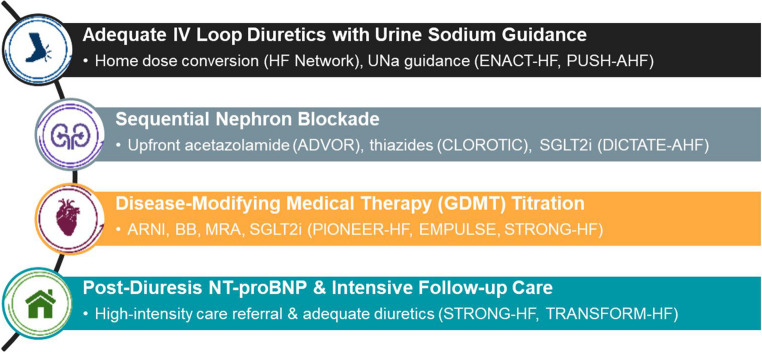
Evidence-Based Approach to Acute Heart Failure Management Key: ADVOR – Acetazolamide in Decompensated Heart Failure with Volume Overload; AHF – Acute Heart Failure; ARNI – Angiotensin Receptor–Neprilysin Inhibitor; BB – Beta-blocker; CLOROTIC – Safety and Efficacy of the Combination of Loop with Thiazide-type Diuretics in Patients with Decompensated Heart Failure; DICTATE-AHF – Efficacy and Safety of Dapagliflozin in Acute Heart Failure; ENACT-HF – Efficacy of a Standardized Diuretic Protocol in Acute Heart Failure; GDMT – Guideline-Directed Medical Therapy; HF – Heart Failure; MRA – Mineralocorticoid Receptor Antagonist; NT-proBNP – Aminoterminal pro–B-type Natriuretic Peptide; PIONEER-HF – Comparison of Sacubitril–Valsartan versus Enalapril on Effect on NT-proBNP in Patients Stabilized from an Acute Heart Failure Episode; PUSH-AHF – Pragmatic Urinary Sodium-based Treatment Algorithm in Acute Heart Failure; SGLT2i – Sodium–Glucose Cotransporter-2 Inhibitor; STRONG-HF – Safety, Tolerability and Efficacy of Rapid Optimization, Helped by NT-proBNP testing, of Heart Failure Therapies; TRANSFORM-HF – Torsemide Comparison with Furosemide for Management of Heart Failure; U_Na_ – Urine Sodium Concentration

In this review, we critically review the growing body of evidence supporting evolving treatment paradigms in acute HF over the past decade and highlight the knowledge gaps that may guide future investigations in three distinctive areas: (1) assessing congestion, kidney dysfunction, and adequacy of diuretic response; (2) ensuring effective decongestion with segmental nephron blockade; and (3) transitioning to GDMT with natriuretic peptide guidance. We also address the emerging importance of post-diuresis NT-proBNP assessment and referral for intensive follow-up care. These domains are integrated in an evidence-based management algorithm summarized in Fig. 1.

## Assessing Congestion, Kidney Dysfunction, and Adequacy of Diuretic Response

### The Problem with Residual Congestion

Adequate decongestion is the clear therapeutic target in hospitalized patients with acute HF. Beyond symptom relief, euvolemia is the physiological foundation that enables every downstream therapy to be tolerated and effective. Nevertheless, defining and quantifying congestion has been challenging as it can manifest either intravascular congestion or tissue congestion or both, each requiring different diagnostic and therapeutic approaches [4, 5]. In the STRONG-HF (Safety, Tolerability and Efficacy of Rapid Optimization, Helped by NT-proBNP Testing, of Heart Failure Therapies) study, successful decongestion was defined as an absence of peripheral edema, pulmonary rales, and jugular venous pressure < 6 cm that can be subjective and may vary among clinicians’ assessments [6]. 

Residual congestion is associated with a heightened risk of early rehospitalization and death, thereby underscoring the need for recognition and intervention [4, 7]. For instance, the efficacy of loop diuretic monotherapy was found to be successful in achieving decongestion in less than 20% of patients in the DOSE trial [8]. However, current methods to assess congestion, including physical examinations, biomarkers, and imaging techniques, are subjective and often not systematically or consistently performed, leading to inconsistent evaluations and variable responses [7]. 

### Kidney Dysfunction in Acute Heart Failure

The reflexive use of loop diuretics, while effective in promptly reducing intravascular congestion, may not address tissue congestion or the underlying cause(s) of congestion and, in turn, promotes diuretic resistance and electrolyte imbalances [4]. [9]. Furthermore, loop diuretics invariably drive up the kidneys’ intrinsic sodium avidity via neurohormonal activation, thereby stimulating sodium reabsorption and leading to further volume overload [10], suboptimal responses to loop diuretics, and incomplete decongestion at discharge [11]. 

### Interpreting Changes in Serum Creatinine and Electrolyte Imbalances

An increase in serum creatinine (sCr, often known as “worsening kidney function”) is frequently observed during diuretic administration in acute HF and is associated with poorer long-term outcomes [12]. However, the interpretation of these changes must be contextualized within the clinical scenario to guide appropriate treatment strategies [12]. Meanwhile, patients with advanced HF often exhibit complex hemodynamic profiles that contribute to renal impairment beyond simple hypoperfusion, including central hemodynamic aberrations [13]. Rise in serum sCr during acute HF or decongestion is a common phenomenon that is often misinterpreted as worsening kidney function. This observed phenomenon can be explained by changes in intraglomerular hemodynamics and intravascular volume depletion that are associated with effective decongestion rather than structural kidney damage or tubular injury [14–16]. Studies suggest that a rise in sCr of up to 0.5 mg/dL from baseline is generally acceptable and may even indicate a positive prognosis if accompanied by effective decongestion (so-called “permissive hypercreatininemia”) [14]. Understanding the nuances of renal sodium management and the implications of (or limiting overreaction to) sCr changes is crucial to avoid residual congestion in patients with incomplete decongestion [17]. Furthermore, the role of electrolyte imbalances in HF progression was further elucidated, with hypochloremia being independently associated with increased mortality risk, even after adjusting for serum sodium levels [18]. This finding underscores the complex interplay between electrolytes and neurohormonal activation in driving sodium avidity and congestion in patients with HF.

### Inadequate Diuretic Dosing Versus True Diuretic Resistance

There is significant variability in diuretic responses among patients hospitalized with acute HF, which can lead to persistent congestion and poor outcomes due to inadequate diuresis [19, 20]. Diuretic resistance often develops over time, thereby further complicating effective decongestion [21]. To improve decongestion outcomes, a defined goal with a consistent diuresis target, especially in the early treatment phase, is necessary. However, real-world diuretic practices often fall short due to delays in initiating and escalating therapy, as well as suboptimal initial dosing strategies [22, 23]. A retrospective analysis of diuretic administration practices in acute HF found substantial delays in achieving maximum diuretic therapy, with a median time to maximum diuretic therapy of 1.8 days, and prolonged delays in diuretic intensification were associated with more extended hospital stays and increased use of adjunctive decongestive therapies [22]. Furthermore, only two-thirds of patients received optimal initial intravenous diuretic therapy, defined as at least two times the home oral loop diuretic dose, especially in those with higher home oral loop diuretic doses [23]. Given these findings, clinicians must ensure that intravenous loop diuretic dosing is optimized at admission, particularly in patients on chronic high-dose diuretics, to prevent early diuretic resistance and avoid unnecessary delays in decongestion.

### Predicting Diuretic Resistance: the BAN-ADHF Score

To address this, one such bedside tool has been developed by compiling 12 readily available clinical variables at the time of hospital admission via a machine-learning algorithm to calculate the risk of diuretic resistance [24]. High BAN-ADHF scores (> 12) were associated with a greater likelihood of inadequate decongestion (defined as urine output < 3 L/day) with intravenous loop diuretics, inadequate dosing, and poorer outcomes [24, 25]. There is also promising pilot data indicating that patients with higher scores may benefit from more intensive diuresis as part of the transition of care to ensure adequate decongestion [26]. 

### The Promise of Natriuretic-Guided Diuresis

Natriuresis, the process of sodium excretion in the urine, offers a measurable and actionable marker for guiding therapy. Specifically, urine sodium excretion has emerged as an objective and clinically relevant marker of response to decongestive therapies, offering prognostic value that surpasses traditional clinical assessments [27]. Patients unable to achieve a negative sodium balance, even in the context of fluid loss, experienced significantly worse outcomes [20]. In addition, low urine sodium levels correlate with increased hospital admissions for acute HF [28]. By providing objective metrics for assessing diuretic response and congestion status, these approaches have the potential to overcome many of the limitations associated with current clinical practices, ultimately leading to improved patient outcomes and reduced rehospitalization rates.

### Linking Natriuresis To Clinical Outcomes

The first piece of evidence came from a prospective study that interrogated the natriuretic responses to continuous intravenous furosemide infusion in patients with acute HF. By directly quantifying urinary excretion of sodium (U_Na_) per measured urine furosemide concentration that reflects the pharmacodynamics of steady-state furosemide administration, Singh et al. linked inadequate natriuresis with inadequate decongestion, worsening kidney function, and adverse long-term outcomes independent of estimated glomerular filtration rate [29]. Specifically, a U_Na_-to-urine furosemide ratio < 2 mmol/mg (corresponding to U_Na_ <50 mmol) had the lowest degree of weight loss and net fluid output over 24 h [29]. Building on these findings, researchers developed and validated an equation to predict net sodium output using a spot urine sample obtained 1–2 h after loop diuretic administration [30, 31]. In their prospective study of 50 patients with acute HF, they found that poor natriuretic response (defined as cumulative U_Na_ output < 50 mmol) could be accurately predicted using their sodium prediction Eq. [31] Confirmed by a recent validation study on oral diuretic dosing as well [32], this approach allows for rapid and highly accurate prediction of diuretic response, potentially enabling early intervention and personalized treatment strategies.

### Identifying Intrinsic Renal Sodium Avidity

Further exploration of intrinsic renal sodium avidity in patients with acute HF was conducted through analysis of baseline data from the ROSE-AHF (Renal Optimization Strategies Evaluation in Acute Heart Failure) trial, in which random UNa spot samples were collected within 24 h of hospital admission, independent of diuretic administration. Those with high sodium avidity (low U_Na_) had less improvement across multiple decongestive metrics, including weight loss, dyspnea relief, decrease in natriuretic peptide levels, and total natriuresis [11]. Patients with high sodium avidity also exhibited more persistent edema and longer hospital stays, highlighting the clinical significance of sodium avidity in acute HF management [11]. Collectively, these findings confirmed the threshold of U_Na_ <50–70 mEq/L in identifying patients with acute HF with higher diuretic needs and poor outcomes.

### Natriuresis-guided Diuretic Dosing and Escalation: ENACT-HF and PUSH-AHF

The ENACT-HF (Efficacy of a Standardized Diuretic Protocol in Acute Heart Failure) study reported significantly higher natriuresis after one day of treatment in the protocol arm compared to the standard care arm [33]. This improved natriuretic response persisted after two days, accompanied by significantly higher diuresis [33]. The study also found that patients in the protocol arm achieved more complete decongestion, highlighting the potential of U_Na_-guided therapy to enhance diuretic efficacy and improve clinical outcomes.

Building upon these findings, the PUSH-AHF (Pragmatic Urinary Sodium-based Treatment Algorithm in Acute Heart Failure) trial further validated the concept of natriuresis-guided diuretic therapy in acute HF and demonstrated that natriuresis-guided therapy significantly improved natriuresis and diuresis up to 48 h without impacting all-cause mortality and/or HF hospitalization at 180 days [34]. Importantly, the natriuresis-guided approach was safe and did not result in renal or electrolyte perturbations despite higher cumulative loop diuretic doses used [34]. 

### Implementing Natriuresis-Guided Protocols

The PUSH-AHF trial also provided insights into the implementation of natriuresis-guided protocols in clinical practice. The study utilized a treatment algorithm that involved spot urinary sodium samples obtained at set time points. If urinary sodium values or diuresis were insufficient (< 70 mmol/L or < 150 mL/h, respectively), decongestive therapy was adjusted based on a prespecified treatment algorithm [34]. This approach resulted in treatment intensification in 85% of patients in the natriuresis-guided group, demonstrating the potential for more aggressive and personalized decongestion strategies [34]. The key clinical studies supporting natriuresis-guided decongestion, including investigations of urine sodium as a prognostic tool and randomized trials such as PUSH-AHF and ENACT-HF, are summarized in Table 1.

**Table 1:** Evidence regarding natriuresis-guided decongestion

Study Name	Study Design	Intervention	Primary Outcome	Key Findings	Contribution to Evidence-Based Management of Acute HF
Insufficient Natriuretic Response to Continuous Intravenous Furosemide in Acute HF[29]	Single-center, prospective cohort study of 52 patients at Cleveland Clinic.	Continuous intravenous furosemide infusion with measurement of urine sodium (UNa) and urine furosemide (UFurosemide).	Association of natriuretic response with worsening kidney function and adverse long-term outcomes.	Lower UNa: UFurosemide ratio correlated with higher likelihood of worsening kidney function and adverse clinical outcomes. Poor natriuretic response was linked to inadequate decongestion and increased long-term mortality, heart failure rehospitalization, and cardiac transplantation.	Highlights the importance of monitoring urine sodium excretion as a predictor of diuretic response and long-term outcomes in acute HF. Suggests potential clinical utility for urine sodium profiling to guide loop diuretic therapy.
Natriuretic Response Is Highly Variable and Associated With 6-Month Survival: Insights From the ROSE-AHF Trial[20]	Post-hoc analysis of the ROSE-AHF trial (*n* = 316) focusing on urinary sodium excretion and its prognostic value in Acute HF patients receiving high-dose loop diuretics.	24-hour urine sodium excretion measurements during high-dose IV loop diuretic therapy; patients classified based on natriuretic response.	Association between urinary sodium excretion and 6-month survival.	Urinary sodium excretion was highly variable and was not correlated with diuretic dose. Higher sodium excretion was associated with reduced 6-month mortality, while traditional fluid-based metrics (urine output, net fluid balance, weight change) were not significant predictors of survival. Patients with positive sodium balance (< 2 g excretion) had worse survival even if net fluid balance was negative.	Suggests that direct measurement of urinary sodium may provide better prognostic and therapeutic value in acute HF than fluid-based metrics. Emphasizes the need for individualized sodium monitoring to optimize diuretic therapy and improve outcomes in acute HF patients.
Rapid and Highly Accurate Prediction of Poor Loop Diuretic Natriuretic Response in Patients With Heart Failure[31]	Single-center, prospective cohort study of 50 patients at Yale New Haven Hospital.	Spot urine sample collected 1–2 h after loop diuretic administration to predict natriuretic response.	Ability of the sodium prediction equation to accurately predict poor diuretic response (≤ 50 mmol sodium excretion in 6 h).	The sodium prediction equation had excellent accuracy (AUC = 0.95) in predicting poor natriuretic response. It outperformed net fluid output and weight loss as measures of diuretic effectiveness.	Potential to improve diuretic monitoring and dosing decisions in acute decompensated heart failure, reducing hospital length of stay and optimizing decongestion strategies.
A Phenomapping Tool and Clinical Score to Identify Low Diuretic Efficiency in Acute Decompensated Heart Failure (BAN-ADHF)[24]	Pooled analysis of AHF trials (ROSE-AHF, CARRESS-HF, ATHENA-HF) to derive (*n* = 794) and externally validate (DOSE/ESCAPE, *n* = 309) a risk score.	Development and validation of an integer-based risk score (BAN-ADHF) using machine learning to predict diuretic efficiency based on baseline clinical variables.	Association of the BAN-ADHF score with diuretic efficiency, length of stay, and in-hospital mortality.	The BAN-ADHF score strongly predicted diuretic efficiency (C-index: 0.92 in validation cohort), outperforming individual markers like creatinine or NT-proBNP. Higher scores were associated with lower diuretic efficiency, longer hospital stays, and higher in-hospital mortality.	Provides a validated, easy-to-use clinical tool to identify patients with diuretic resistance at presentation, allowing for proactive, risk-stratified decongestive strategies rather than a reactive, trial-and-error approach.
Natriuresis-guided diuretic therapy in acute heart failure: a pragmatic randomized trial: PUSH-AHF[34]	Pragmatic, open-label, randomized controlled trial of 310 patients at University Medical Center Groningen, Netherlands.	Natriuresis-guided diuretic therapy with intensified diuretic treatment based on spot urinary sodium levels vs. standard of care.	24-hour urinary sodium excretion and time to all-cause mortality or heart failure rehospitalization at 180 days.	Natriuresis-guided therapy improved 24-hour natriuresis (409 ± 178 mmol vs. 345 ± 202 mmol in SOC, *P* = 0.0061), but did not significantly reduce mortality or rehospitalization.	Supports the use of natriuresis as a marker for optimizing diuretic therapy, potentially informing personalized treatment strategies for acute HF patients.
Protocolized Natriuresis-Guided Decongestion Improves Diuretic Response: The Multicenter ENACT-HF Study[33]	Nonrandomized, open-label, multicenter, pragmatic study conducted at 29 centers in 18 countries with 401 patients.	Standardized natriuresis-guided diuretic protocol vs. standard of care.	Natriuresis after 1 day.	Natriuresis was significantly higher in the protocol arm (282 vs. 174 mmol; *P* < 0.001), with higher diuresis and a shorter length of stay (5.8 vs. 7.0 days; *P* = 0.036). In-hospital mortality was similar in both arms.	Supports the use of urinary sodium to guide decongestion in acute HF, with a protocol that may be safely implemented across diverse healthcare settings to improve diuretic response.

Key: *AHF* Acute Heart Failure; *AUC* Area Under the Curve; *BAN-ADHF* Blood Urea Nitrogen, Creatinine, Natriuretic Peptide Levels, Atrial Fibrillation, Diastolic Blood Pressure, Hypertension. Home Diuretic use, and Heart Failure Hospitalization (risk score); *CARRESS-HF* Cardiorenal Rescue Study in Acute Decompensated Heart Failure; *ENACT-HF* Efficacy of a Standardized Diuretic Protocol in Acute Heart Failure; *ESCAPE* Evaluation Study of Congestive Heart Failure and Pulmonary Artery Catheterization Effectiveness; *HF* Heart Failure; *IV* Intravenous; *NT-proBNP* aminoterminal pro–B-type Natriuretic Peptide; *PUSH-AHF* Pragmatic Urinary Sodium-based Treatment Algorithm in Acute Heart Failure; *ROSE-AHF* Renal Optimization Strategies Evaluation in Acute Heart Failure; *SOC* Standard of Care; *UFurosemide* Urine Furosemide Concentration; *UNa* Urine Sodium Concentration

### Opportunities for Improvement and Implementation into Practice

Frequent reassessment of diuretic efficacy within 2–6 h of administration is essential for achieving optimal decongestion [35]. This is particularly important given that intravenous loop diuretics like furosemide have a relatively short duration of action, typically 2–3 h, necessitating timely dose adjustments to maintain therapeutic efficacy [36]. Establishing the goal of diuretic therapy in acute HF to achieve a net negative fluid balance of 3–5 L daily. Regular assessment of natriuretic response within each clinical shift is crucial, especially during the first 24–48 h of admission, when urine output data is inadequately captured. This is because the short 2–3 h duration of action for loop diuretics requires timely interventions to maintain therapeutic efficacy [35, 36]. To accomplish this, monitoring spot U_Na_ excretion > 50–70 mEq/L within the first 2 h (or even randomly) or a urine output > 100–150 mL/hour within the first 6 h following intravenous diuretic administration, predicts an adequate diuretic response and can help guide dose escalation when necessary [1]. This approach helps prevent delays in treatment modification and ensures optimal decongestion outcomes. Ultimately, integrating strategies to mitigate delayed diuretic administration and improve dosing—particularly in patients on high-dose home diuretics—is likely to translate into improved decongestion, reduced length of stay, and better patient outcomes [22, 23]. 

## Ensuring Effective Decongestion with Segmental Nephron Blockade

### Rationale for Multi-Nephron Segment Diuretic Therapy

Current single-agent strategies fail to address the full complexity of nephron segment reabsorption mechanisms, which are crucial for effective fluid management in patients with acute HF. The nephron’s multiple segments, each playing a unique role in sodium and water reabsorption, require a more comprehensive approach than traditional monotherapy can offer. Targeting multiple nephron segments simultaneously can overcome counter-regulatory mechanisms and improve decongestion. Multi-nephron segment diuretic therapy (MSDT), which combines different classes of diuretics, has shown promise in overcoming diuretic resistance. A study demonstrated that MSDT, which includes carbonic anhydrase inhibitors, loop diuretics, thiazides, and mineralocorticoid receptor antagonists, significantly increased urine output and weight loss in patients with severe diuretic resistance without adversely affecting serum chemistries or kidney function [37]. Collectively, these clinical trials evaluating segmental nephron blockade strategies are summarized in Table 2.

**Table 2:** Evidence regarding segmental nephron blockade and additional pharmacologic adjuncts

Study Name	Study Design	Intervention	Primary Outcome	Key Findings	Contribution to Evidence-Based Management of Acute HF
Diuretic Strategies in Patients with Acute Decompensated Heart Failure (DOSE-AHF) [38]	Prospective, double-blind, randomized trial of 308 patients with acute decompensated heart failure.	Patients received furosemide either via bolus every 12 h or continuous infusion, and at either a low dose (oral equivalent) or a high dose (2.5 times oral dose).	Patients’ global assessment of symptoms (AUC of visual-analog scale over 72 h) and change in serum creatinine from baseline to 72 h.	No significant differences in symptoms or renal function between bolus vs. continuous infusion or high-dose vs. low-dose strategies. High-dose strategy led to greater diuresis but transient worsening of renal function.	Findings suggest that continuous infusion offers no clear benefit over bolus dosing, and high-dose strategies may improve diuresis but require monitoring of renal function.
Combining Loop with Thiazide Diuretics for Decompensated Heart Failure: The CLOROTIC Trial [39]	Multicenter, prospective, randomized, double-blind, placebo-controlled trial of 230 patients with acute heart failure.	Patients randomized to receive hydrochlorothiazide (HCTZ) or placebo in addition to an intravenous furosemide regimen for 5 days.	Changes in body weight and patient-reported dyspnea 72 h after randomization.	HCTZ led to greater weight loss at 72 h (−2.3 vs. −1.5 kg, *P* = 0.002) and increased diuresis but did not significantly improve dyspnea. More frequent renal impairment was observed in the HCTZ group (46.5% vs. 17.2%, *P* < 0.001).	Supports the use of combination diuretic therapy for fluid overload but highlights the need for monitoring renal function due to potential worsening with HCTZ.
Acetazolamide in Acute Decompensated Heart Failure with Volume Overload: The ADVOR Trial [40]	Multicenter, randomized, parallel-group, double-blind, placebo-controlled trial of 519 patients at 27 sites in Belgium.	Intravenous acetazolamide (500 mg once daily) added to intravenous loop diuretics.	Successful decongestion, defined as the absence of signs of volume overload within 3 days after randomization without the need for escalation of decongestive therapy.	Acetazolamide led to a higher incidence of successful decongestion (42.2% vs. 30.5% in placebo, *P* < 0.001), with improved diuretic efficiency and no increase in adverse events.	Supports the use of acetazolamide to enhance diuretic efficiency in acute decompensated heart failure with volume overload, potentially improving patient outcomes through more rapid and effective decongestion.
Efficacy and Safety of Dapagliflozin in Patients With Acute Heart Failure (DICTATE-AHF) [41]	Multicenter, open-label, randomized trial of 240 patients with hypervolemic AHF.	Dapagliflozin 10 mg once daily vs. structured usual care with protocolized diuretic titration.	Diuretic efficiency, expressed as cumulative weight change per cumulative loop diuretic dose at 5 days.	The primary outcome was not met (OR: 0.65; 95% CI: 0.41–1.02; *P* = 0.06). However, the dapagliflozin group required significantly lower loop diuretic doses to achieve similar weight loss (median 560 mg vs. 800 mg, *P* = 0.006) and had greater 24-hour natriuresis and diuresis.	Demonstrates a diuretic-sparing effect of dapagliflozin, reframing SGLT2i initiation in AHF as both an acute decongestive strategy and initiation of long-term GDMT. Supports early initiation for dual benefit.
Rationale and Design of the DECONGEST (Diuretic Treatment in Acute Heart Failure With Volume Overload Guided by Serial Spot Urine Sodium Assessment) Study [42]	Pragmatic, 2-center, randomized, open-label study aiming to enroll 104 patients with AHF.	An intensive, bundled diuretic regimen (thrice-daily IV boluses, early combination therapy) guided by serial spot urine sodium assessments vs. standard of care.	Hierarchical composite of 30-day survival, days alive and out of hospital, and relative decrease in natriuretic peptides.	Trial is ongoing; results are not yet available. The study is designed to test if a highly structured, aggressive, natriuresis-guided protocol improves clinical outcomes.	Rationale and design published. Aims to determine if linking natriuresis measurement to a potent, non-discretionary therapeutic algorithm can translate physiological improvements into better clinical outcomes, addressing a key gap from prior studies.
Oral Sodium to Preserve Renal Efficiency in Acute Heart Failure: A Randomized, Placebo-Controlled, Double-Blind Study (OSPREY-AHF) [43]	Single-center, randomized, double-blind, placebo-controlled trial of 70 patients with AHF.	Oral sodium chloride (2 g three times daily) vs. placebo, in addition to continuous IV furosemide infusion.	Bivariate endpoint of change in serum creatinine and change in weight at 96 h.	No significant difference between groups for the primary endpoint (*P* = 0.33). No differences in weight change, creatinine change, or other efficacy/safety endpoints were observed.	Provides evidence that a liberal oral sodium strategy does not improve diuretic efficiency or protect renal function during aggressive diuresis, challenging the sodium supplementation hypothesis and reinforcing focus on pharmacologic strategies.
Efficacy and safety of hypertonic saline therapy in ambulatory patients with heart failure: The SALT-HF trial [44]	Multicenter, double-blind, randomized trial of 167 ambulatory patients with worsening HF.	Single 1-hour infusion of IV furosemide plus hypertonic saline solution (HSS) vs. IV furosemide plus placebo.	Volume of diuresis at 3 h.	No difference in the primary endpoint of 3-hour diuresis (1099 mL vs. 1103 mL, *P* = 0.963). No differences in 3-hour natriuresis, weight change, or 30-day clinical events.	Demonstrates that adding a single infusion of HSS to IV furosemide does not augment acute diuresis in ambulatory worsening HF, providing further evidence against the utility of sodium-loading strategies for decongestion.

Key: *ADVOR* Acetazolamide in Decompensated Heart Failure with Volume Overload; *AHF* Acute Heart Failure; *AUC* Area Under the Curve; *CI* Confidence Interval; *CLOROTIC* Safety and Efficacy of the Combination of Loop with Thiazide-type Diuretics in Patients with Decompensated Heart Failure; *DECONGEST* Diuretic Treatment in Acute Heart Failure with Volume Overload Guided by Serial Spot Urine Sodium Assessment; *DICTATE-AHF* Efficacy and Safety of Dapagliflozin in Acute Heart Failure; *DOSE-AHF* Determining Optimal Dose and Duration of Diuretic Treatment in People with Acute Heart Failure; *GDMT* Guideline-Directed Medical Therapy; *HCTZ* Hydrochlorothiazide; *HF* Heart Failure; *HSS* Hypertonic Saline Solution; *IV* Intravenous; *OR* Odds Ratio; *OSPREY-AHF* Oral Sodium to Preserve Renal Efficiency in Acute Heart Failure; *SALT-HF* Efficacy and Safety of Hypertonic Saline Therapy in Ambulatory Patients with Heart Failure; *SGLT2i* Sodium–Glucose Cotransporter-2 Inhibitor

### Distal Nephron Blockade in Acute HF: the CLOROTIC and ATHENA-HF Trials

The CLOROTIC (Safety and Efficacy of the Combination of Loop with Thiazide-type Diuretics in Patients with Decompensated Heart Failure) trial focused on the role of thiazide diuretics in blocking distal nephron sodium reabsorption. It demonstrated that adding hydrochlorothiazide (HCTZ) to intravenous furosemide improved diuretic response in acute HF patients. Those receiving HCTZ had greater weight loss at 72 h and increased 24-hour diuresis compared to placebo [39]. However, the trial noted more frequent impairment of renal function with HCTZ and electrolyte imbalance, highlighting the need for careful patient monitoring [39].

While low-dose mineralocorticoid receptor antagonist (MRA) therapy is well-established for chronic HF, the role of high-dose MRA therapy in acute HF remains uncertain. However, the ATHENA-HF (Aldosterone Targeted Neurohormonal Combined with Natriuresis Therapy in Heart Failure) trial did not find a significant improvement in natriuretic peptide levels or clinical congestion with high-dose spironolactone in patients with acute HF already receiving intravenous loop diuretics, even in those at higher risk with kidney impairment [45, 46]. This may be in part due to the delayed actions as a result of conversion to active metabolite, canrenone [47], but add-on spironolactone can limit potassium wasting, potentially reducing the need for potassium supplementation [48]. 

### Proximal Nephron Blockade: the ADVOR Trial

The ADVOR (Acetazolamide in Decompensated Heart Failure with Volume Overload) trial investigated acetazolamide, a carbonic anhydrase inhibitor, to inhibit sodium reabsorption in the proximal tubule. The study demonstrated improved decongestion within three days and at discharge for patients with elevated natriuretic peptides [40]. Acetazolamide use was associated with a shorter hospital stay and did not increase side effects except for higher 90-day hypokalemia rates [40]. The incidence of adverse events, including hypokalemia, was similar between the two groups, indicating that acetazolamide did not significantly increase side effects. These findings suggest that acetazolamide could serve as an effective adjunct therapy to intravenous loop diuretics, potentially mitigating neurohormonal activation and enhancing overall decongestion efficacy. The beneficial effects of acetazolamide were observed across the spectrum of left ventricular ejection fraction and baseline renal function. Further analysis of the ADVOR trial revealed that acetazolamide’s effectiveness was particularly pronounced in patients with elevated baseline bicarbonate levels (≥ 27 mmol/L), indicating a state of neurohormonal activation and increased proximal sodium reabsorption [49]. In these patients, acetazolamide significantly improved decongestive response, diuretic efficiency, and reduced length of stay. The study also found that acetazolamide prevented the progressive increase in bicarbonate levels typically observed with loop diuretic monotherapy, suggesting its ability to counteract diuretic resistance [49]. 

Additionally, acetazolamide demonstrated a strong and independent effect on natriuresis, increasing urine sodium concentration by 19% and total natriuresis by 32%[50]. This enhanced natriuretic response was associated with faster and more complete relief of volume overload signs, translating into shorter hospital stays. Notably, every 10 mmol/L increase in urine sodium concentration was independently associated with a lower risk of all-cause death or HF readmission, highlighting the potential prognostic value of natriuresis in acute decompensated HF management [50]. 

### Salt Supplementation

Adequate sodium delivery to the distal nephron is necessary for loop diuretic effectiveness, effective natriuresis, and preventing counterproductive sodium avidity. The concept of salt supplementation in acute HF challenges traditional assumptions and is grounded in evolving insights into the pathophysiology of neurohormonal activation, diuretic resistance, and intravascular depletion. In two contemporary pilot studies with intravenous hypertonic saline as well as oral sodium chloride (~ 3–6 g/day) given to diuretic-resistant patients with acute HF, targeted sodium repletion may optimize decongestion and improve renal safety [43, 44]. Post-hoc analysis further imply that adding hypertonic saline to hypochloremic patients may improve short-term natriuretic response and outcomes, and warrants further investigation [51]. 

### Opportunities for Improvement and Implementation into Practice

Based on the evidence from ADVOR and CLOROTIC trials, adopting a combined nephron-targeting approach, integrating acetazolamide or thiazides with loop diuretics, appears promising to enhance decongestion in patients with acute HF. This strategy has shown promise in overcoming diuretic resistance, as evidenced by increased urine output and weight loss without significant changes in serum chemistries or kidney function [8, 37]. Prioritizing the early use of adjunctive therapies, such as acetazolamide, during the critical first three days can further improve patient outcomes by ensuring more effective natriuresis and fluid removal [35]. Close monitoring of electrolyte levels, including sodium, potassium, and chloride, is essential to mitigate the risks of imbalances, particularly when using thiazides [8, 37]. Regular assessment of renal function and urine output is crucial to dynamically tailor therapy and prevent potential complications such as acute renal failure [37, 52]. 

## Transitioning To GDMT with Natriuretic Peptide Guidance

### The Post-Stabilization Gap in GDMT Initiation

Patients hospitalized with acute HF are often discharged without the initiation of GDMT, missing a critical window following stabilization. The gap in care is evident as reflected through various studies, which highlight that a significant proportion of patients do not receive optimal doses of GDMT at discharge. It is estimated that only a third of patients were on optimal doses of GDMT at discharge, with the majority not optimized, which is associated with high readmission rates [53]. In a study involving 3,164 hospitalized patients with acute HF, only 17.1% received GDMT agents up-titrated to more than 50% of the maximum titrated dose at discharge, despite 75% being eligible for such therapy [54]. Studies have shown that initiating GDMT in the hospital setting is associated with better adherence and optimization post-discharge [55]. However, variability in provider practices and the lack of standardized protocols contribute to the underutilization of GDMT [56]. Clinical dashboards and problem visualization tools can help identify patients who need GDMT optimization and secure buy-in from healthcare providers to implement changes [57]. Pharmacist-led medication titration has been shown to facilitate more rapid up-titration of GDMT, reducing hospital readmissions [53]. 

### In-Hospital ARNI Initiation: the PIONEER-HF Trial

The introduction of angiotensin receptor-neprilysin inhibitors (ARNIs) has marked a significant advancement in HF treatment. In the PIONEER-HF (Comparison Of Sacubitril/valsartan Versus Enalapril on Effect on NT-pro-BNP in Patients Stabilized From an Acute Heart Failure Episode) trial, patients with acute HF and elevated natriuretic peptides (> 1,500 pg/mL) randomized to sacubitril/valsartan while still admitted to the hospital had a greater reduction in aminoterminal pro-B-type natriuretic peptide (NT-proBNP) levels from baseline to weeks 4 and 8 compared to those receiving enalapril, with trends towards improved clinical outcomes [58]. 

### The Role of SGLT2 Inhibitors: the EMPULSE Trial

Meanwhile, the EMPULSE (Empagliflozin in Patients Hospitalized With Acute Heart Failure Who Have Been Stabilized) trial provided compelling evidence for the efficacy of SGLT2 inhibitors in improving clinical outcomes during hospitalization and beyond, and empagliflozin initiated during the in-hospital phase led to significant clinical benefit [59]. The primary outcome, a clinical benefit score based on death, HF events, time to first HF event, and change in Kansas City Cardiomyopathy Questionnaire Total Symptom Score at 90 days, was significantly in favor of empagliflozin [59]. Further analysis of the EMPULSE trial found that empagliflozin led to significantly greater reductions in all studied markers of decongestion [60]. Weight loss was significantly greater in the empagliflozin group at days 15, 30, and 90. NT-proBNP levels and clinical congestion scores also showed greater reductions in the empagliflozin group [60]. Importantly, greater weight loss at day 15 was associated with a higher probability of clinical benefit at day 90, suggesting that early and effective decongestion may contribute to improved outcomes in patients with acute HF.

### In-Hospital SGLT2 Inhibitors: DAPA ACT HF–TIMI 68 and Prespecified Meta-analysis

Beyond symptom and decongestion signals, larger outcome-focused data further inform the class effect of early SGLT2 inhibitor initiation. The Dapagliflozin Effect on Cardiovascular Events in Acute Heart Failure (DAPA ACT HF–TIMI 68) trial, the largest randomized study to date in this setting (*n* = 2,401), evaluated in-hospital initiation of dapagliflozin in stabilized patients with acute HF across the spectrum of ejection fraction [61, 62]. The primary efficacy endpoint, time to cardiovascular death or first worsening HF event through two months, was not met; over approximately 60 days, dapagliflozin did not significantly reduce the composite versus placebo (*p* = 0.20)[61]. This neutral result must be interpreted in the context of the trial’s design, which included a short follow-up duration, a high proportion of de novo HF (~ 45%) who have a better short-term prognosis, and a lower-than-anticipated event rate, collectively limiting statistical power [61, 62]. Importantly, dapagliflozin was associated with a clinically meaningful trend toward lower all-cause mortality (3.0% vs. 4.5% with a reassuring safety profile, including low rates of symptomatic hypotension (3.6% vs. 2.2%) and worsening kidney function (5.9% vs. 4.7%)[61].

The trial’s prespecified meta-analysis pooled DAPA ACT HF–TIMI 68 with EMPULSE and the in-hospital cohort of SOLOIST-WHF (total *n* = 3,527) and demonstrated significant early reductions in both the composite of cardiovascular death or worsening HF (*p* = 0.012) and in all-cause mortality (*p* = 0.001), thereby resolving the statistical ambiguity of the single neutral trial and reinforcing a class effect for early initiation [61]. These pooled findings are consistent with the broader SGLT2 inhibitor evidence base and the vulnerable post-discharge risk window [63, 64], supporting the concept that starting therapy before discharge improves near-term outcomes. Operationally, SGLT2 inhibitors also integrate into decongestive care pathways. In a contemporary network meta-analysis of add-on diuretic strategies in acute HF, SGLT2 inhibitors ranked highly for shorter hospital length of stay (second only to acetazolamide by SUCRA), consistent with improved diuretic efficiency with no signal of excess renal or electrolyte harm; however, these indirect comparisons should be interpreted cautiously [65]. Taken together, the totality of evidence supports the feasibility, safety, and likely mortality benefit of initiating SGLT2 inhibitors prior to discharge in stabilized acute HF patients—aligning with high-intensity, protocol-driven strategies that prioritize getting foundational therapy on board before patients leave the hospital.

### High-Intensity Care Strategy: the STRONG-HF Trial

The ultimate demonstration of the benefits of in-hospital GDMT initiation and uptitration came from the STRONG-HF study, which demonstrated that a high-intensity care strategy involving rapid up-titration of GDMT was associated with improved survival and reduced hospital readmissions [66]. This protocol-driven study for those with persistently elevated NT-proBNP that emphasized initiation of all GDMT at the time of discharge with weekly follow-up with enhanced diuresis also demonstrated the safety of rapid uptitration of GDMT following stabilization and intensive weekly follow-up of patients hospitalized with acute HF [67, 68]. Furthermore, persistent congestion following discharge was significantly reduced in the high-intensity care arm that was associated with a lower risk of 180-day HF readmission or all-cause death, underscoring the importance of early and sustained GDMT despite being on a lower mean daily dose of loop diuretics [6]. The GDMT intensity score at baseline was < 6 in 358 (33%) patients, 6 to 7 in 329 (31%) patients, and > 7 in 386 (36%) patients. At 90 days, 88.4% of patients in the high-intensity arm achieved a score > 7 versus 14.3% in the usual care arm (*p *< 0.0001)[69].

#### Key Lessons from the STRONG-HF Protocol

The STRONG-HF study highlights three key points in the management of decongestion in acute HF: (1) the introduction of GDMT, after initial management with intravenous diuretics, may already start in the emergency room with the start of full dose of SGLT2 inhibitors and half dose of MRAs, if possible; (2) the combination of the four GDMT drugs should be initiated during the index hospital stay; and (3) all efforts should be made to achieve full doses of the four pillars of HF within 6 weeks [70]. Together, these findings from pivotal randomized trials, including STRONG-HF, EMPULSE, PIONEER-HF, and ATHENA-HF, provide the foundation for current recommendations on early initiation and maintenance of disease-modifying therapies in acute heart failure, with key details summarized in Table 3.

**Table 3:** Maintenance and early initiation of disease-modifying therapies

Study Name	Study Design	Intervention	Primary Outcome	Key Findings	Contribution to Evidence-Based Management of Acute HF
Safety, tolerability and efficacy of up-titration of guideline-directed medical therapies for acute heart failure (STRONG-HF): a multinational, open-label, randomized, trial [66]	Multinational, open-label, randomized, parallel-group trial conducted in 87 hospitals across 14 countries with 1078 patients.	High-intensity care (rapid up-titration of guideline-directed therapy within 2 weeks + close follow-up) vs. usual care.	180-day all-cause death or heart failure readmission.	High-intensity care led to significantly lower rates of heart failure readmission or all-cause death (15.2% vs. 23.3%, *p* = 0.0021) and greater reductions in NT-proBNP, NYHA class, and congestion markers, though with a higher rate of non-serious adverse events.	Supports the safety and efficacy of early, intensive up-titration of guideline-directed HF therapies post-hospitalization with frequent monitoring to reduce heart failure readmissions and mortality. May potentially shape future HF management guidelines by emphasizing early intervention and structured follow-up.
The SGLT2 inhibitor empagliflozin in patients hospitalized for acute heart failure (EMPULSE) [59]	Multinational, double-blind, randomized controlled trial of 530 patients across 118 centers in 15 countries.	Empagliflozin 10 mg once daily vs. placebo in hospitalized acute heart failure patients.	Composite clinical benefit at 90 days (all-cause death, heart failure events, and symptom improvement assessed via win ratio).	Empagliflozin resulted in a superior clinical benefit (win ratio: 1.36; *P* = 0.0054). All-cause mortality was lower (4.2% vs. 8.3%), and heart failure events occurred less frequently (10.6% vs. 14.7%) compared to placebo.	Supports initiation of empagliflozin in hospitalized acute HF patients, reinforcing SGLT2 inhibitors as an effective treatment for both de novo and decompensated heart failure.
Angiotensin–Neprilysin Inhibition in Acute Decompensated Heart Failure (PIONEER-HF) [71]	Multicenter, randomized, double-blind, active-controlled trial of 881 patients at 129 U.S. hospitals.	Sacubitril–valsartan vs. Enalapril for patients hospitalized for acute decompensated heart failure.	Time-averaged proportional change in NT-proBNP concentration from baseline through weeks 4 and 8.	Sacubitril–valsartan showed a greater reduction in NT-proBNP compared to enalapril (−46.7% vs. −25.3%, *P* < 0.001); no significant difference in safety outcomes (renal function, hyperkalemia, symptomatic hypotension, angioedema).	Demonstrates that initiation of sacubitril–valsartan in hospitalized acute decompensated heart failure patients is safe and results in a greater NT-proBNP reduction compared to enalapril. Supports the use of sacubitril–valsartan early in treatment for improved outcomes.
Efficacy and Safety of Spironolactone in Acute Heart Failure: The ATHENA-HF Randomized Clinical Trial [45]	Double-blind, randomized, placebo/low-dose controlled trial of 360 AHF patients at 22 U.S. hospitals.	High-dose spironolactone (100 mg daily) vs. usual care (placebo or 25 mg spironolactone) for 96 h.	Proportional change in NT-proBNP from baseline to 96 h.	No significant difference in NT-proBNP reduction between groups (*P* = 0.57). No improvement in secondary endpoints of congestion, urine output, or weight change. High-dose spironolactone was well-tolerated with no excess hyperkalemia or renal dysfunction.	Clarifies that high-dose spironolactone does not function as an effective acute diuretic in AHF. However, its demonstrated safety profile removes a key barrier to initiating or continuing guideline-recommended MRA therapy during hospitalization for its proven long-term benefits.
Dapagliflozin and Effect on Cardiovascular Events in Acute Heart Failure -Thrombolysis in Myocardial Infarction 68 **(**DAPA ACT HF-TIMI 68) [61]	Multinational, double-blind, randomized, placebo-controlled trial at 210 sites in 5 countries with 2,401 hospitalized HF patients; randomized ≥ 24 h to ≤ 14 d after admission once stabilized; 60-day treatment and follow-up.	Dapagliflozin 10 mg once daily initiated in-hospital vs. matching placebo.	Time to cardiovascular death or first worsening HF event through 2 months.	Primary endpoint neutral (10.9% vs. 12.7%; *p* = 0.20); all-cause death numerically lower (3.0% vs. 4.5%); prespecified meta-analysis of in-hospital SGLT2 inhibitor trials (DAPA ACT HF–TIMI 68, EMPULSE, SOLOIST-WHF inpatient subgroup; *n* = 3,527) showed significant reductions in cardiovascular death or worsening HF (*p* = 0.012) and all-cause death (*p* = 0.001).	Largest inpatient SGLT2 inhibitor RCT; despite a neutral primary result, pooled evidence supports initiating an SGLT2 inhibitor during HF hospitalization once hemodynamically stable to lower early post-discharge events, with attention to symptomatic hypotension and kidney function monitoring.

Key: *AHF* Acute Heart Failure; *ATHENA-HF* Aldosterone Targeted Neurohormonal Combined with Natriuresis Therapy in Heart Failure; *DAPA ACT HF–TIMI 68* Dapagliflozin and Effect on Cardiovascular Events in Acute Heart Failure–Thrombolysis In Myocardial Infarction 68; *EMPULSE* A Study to Test the Effect of Empagliflozin in Patients Who Are in Hospital for Acute Heart Failure; *HF *Heart Failure; *MRA* Mineralocorticoid Receptor Antagonist; *NT-proBNP* Aminoterminal pro–B-type Natriuretic Peptide; *NYHA* New York Heart Association (functional classification); *PIONEER-HF* Comparison of Sacubitril–Valsartan versus Enalapril on Effect on NT-proBNP in Patients Stabilized from an Acute Heart Failure Episode; *SGLT2* Sodium–Glucose Cotransporter-2; *SOLOIST-WHF *Effect of Sotagliflozin on Cardiovascular Events in Participants With Type 2 Diabetes Post Worsening Heart Failure; *STRONG-HF* Safety, Tolerability and Efficacy of Rapid Optimization, Helped by NT-proBNP Testing, of Heart Failure Therapies

### Natriuretic Peptide-Guided Transition of Care: STRONG-HF and GUIDE-IT Studies

Persistent congestion may also preclude GDMT initiation or uptitration due to concerns about renal impairment, hypotension, and other comorbidities [72]. Traditional methods of assessing discharge readiness, such as changes in weight and physical examination, have proven insufficient, as evidenced by the persistently high rehospitalization rates within 30 days of discharge [73]. As persistent congestion is associated with poorer outcomes, patients who achieve a substantial decrease in NT-proBNP levels during in-hospital treatment (often > 30% from baseline) experienced better outcomes, suggesting that these biomarkers can be effective tools for guiding therapy [73]. Often overlooked is the 2022 AHA/ACC/HFSA clinical guideline endorsement of assessing follow-up or pre-discharge NT-proBNP monitoring [74]. Indeed, persistent elevation of NT-proBNP levels > 1,500 pg/mL 48–72 h following stabilization with loop diuretics was a frequent threshold for recent clinical trials that support in-hospital GDMT titration.

### Using NT-proBNP To Guide Diuresis and Titration

Regular monitoring of NT-proBNP levels facilitates targeted therapy adjustments, potentially reducing the risk of rehospitalization. While the STRONG-HF often emphasized GDMT initiation and intensive uptitration, patients with increased NT-proBNP levels received more diuretics and were up-titrated more slowly during the first weeks after discharge per protocol. A sub-analysis of the STRONG-HF study demonstrated that a > 10% decrease in NT-proBNP levels from admission to pre-discharge. The primary endpoint of 180-day all-cause death or HF readmission occurred in 15.2% of patients in the high-intensity care strategy compared to 23.3% in the usual care group [66]. Often overlooked in the STRONG-HF study is that the same proportion (almost half) of patients in both arms of the experienced successful decongestion at baseline/discharge that was associated with a lower risk of 180-day HF readmission or all-cause death (HR: 0.40; 95% CI: 0.27–0.59; *p* < 0.0001), while those in the high-intensity care arm had a significantly better chance of sustaining decongestion at day 90 [6]. Patients in the high-intensity care group with stable or increased NT-proBNP were older, with more severe acute HF and worse renal and liver function, and with poorer outcomes and less likely to reach optimal GDMT doses compared to those with NT-proBNP decrease [75]. 

### Post-Discharge Monitoring: the GUIDE-IT Trial

Natriuretic peptide-guided management strategy extends to the post-discharge setting. In a comprehensive analysis of the GUIDE-IT (Guiding Evidence Based Therapy Using Biomarker Intensified Treatment in Heart Failure) trial that included 638 patients with HF with reduced ejection fraction, patients achieving an NT-proBNP level ≤ 1,000 pg/mL by 90 days after randomization was associated with significantly better clinical outcomes and better quality of life that those that did not [2]. Importantly, these improved outcomes were observed regardless of baseline NT-proBNP concentrations, suggesting that the achievement of lower NT-proBNP levels during treatment is a crucial factor in prognosis [2]. 

## Predicting Nonresponse with Biomarker Trajectories

At the other end of the spectrum are those with persistently elevated NT-proBNP levels. Post-hoc analysis of the PROTECT study focusing on NT-proBNP “nonresponse” during HF management (NT-proBNP > 1000 pg/mL) found that a decreasing NT-proBNP was associated with better clinical outcomes, while a rising NT-proBNP correlated with increased cardiovascular event rates [76]. The PROTECT (Pro-B Type Natriuretic Peptide Outpatient Tailored Chronic Heart Failure Therapy) study also demonstrated that NT-proBNP-guided HF management was associated with greater improvements in echocardiographic parameters of cardiac structure and function compared to standard care [77]. The researchers developed and validated a risk model for predicting nonresponse [76], which has recently been externally validated in the GUIDE-IT trial [78]. 

### Toward Multi-Marker Strategies

The question regarding whether multiple biomarkers beyond NT-proBNP can help guide post-discharge therapy better has been addressed by the TRIUMPH (Translational Initiative on Unique and Novel Strategies for Management of Patients With Heart Failure) study. The study found that the average estimated NT-proBNP level increased before the primary endpoint (all-cause mortality and HF rehospitalization) was reached, suggesting that regular monitoring could provide early warning signs of clinical deterioration [79]. While the TRIUMPH study also evaluated other biomarkers to investigate the potential of a multi-marker strategy that aligns with the complex pathophysiology of HF, whether this can better facilitate more personalized and precise therapeutic interventions remains to be determined [80]. 

### Bioimpedance for Congestion Monitoring

Beyond serum and urine biomarkers, bioimpedance analysis offers a noninvasive, quantitative method for evaluating fluid status. This technology measures the body’s resistance and reactance to a low-level electrical current, allowing for the estimation of total body water, including its distribution between intracellular and extracellular compartments [81]. In the inpatient setting, techniques such as bioimpedance vector analysis (BIVA) and bioimpedance spectroscopy (BIS) can be used serially to objectively track the response to diuretic therapy [82, 83]. This enables clinicians to guide decongestion toward a defined endpoint of euvolemia and identify patients with prognostically significant residual congestion prior to discharge, which is associated with a higher risk of readmission and mortality [84]. 

The application of bioimpedance extends to the vulnerable post-discharge period, where emerging wearable sensor technologies offer the potential for remote daily monitoring of thoracic or whole-body fluid status [85]. By detecting subclinical fluid accumulation, these systems can provide an early warning of impending decompensation. Preliminary data suggest that changes in transthoracic bioimpedance can predict the need for diuretic intensification or rehospitalization up to a week in advance, creating a crucial window for preemptive intervention to avert clinical deterioration [86]. This technology holds promise for personalizing diuretic management, aiming to optimize fluid status during hospitalization and maintain it long-term to prevent readmissions.

### Maintenance Diuretics: Torsemide Vs. Furosemide: TRANSFORM-HF Trial

The absence of a standardized approach to diuretic management upon discharge can result in significant variability in patient outcomes. For instance, patients discharged from the emergency department or after a brief observation period often do not receive the same level of transitional care as those who are hospitalized, leading to higher rates of readmission and adverse events [87]. The appropriate choice of maintenance diuretics in HF management has been a topic of considerable interest, particularly the comparison between torsemide and furosemide. The TRANSFORM-HF (Torsemide Comparison With Furosemide for Management of Heart Failure) trial, a landmark study in this area, sought to address the long-standing question of whether torsemide offers superior benefits compared to furosemide in HF maintenance therapy [88]. The trial found no significant difference in all-cause mortality between the two diuretics. A subsequent post-hoc analysis further reinforced these findings, demonstrating that the comparative effectiveness of torsemide and furosemide was not influenced by baseline renal function, challenging the traditional preference for torsemide in patients with kidney dysfunction [89]. 

### Mechanistic Insights from TRANSFORM-Mechanism

However, the trial highlighted important considerations regarding bioavailability and potency conversion. While the study protocol suggested a 1:2 to 1:4 conversion ratio (torsemide to furosemide), recent evidence indicates that the oral dose equivalent may be closer to 1:4 [90]. The TRANSFORM-Mechanism trial revealed that torsemide did not demonstrate superior renal tubular delivery or duration of action compared to furosemide, contrary to previous assumptions. The study found that a 4:1 dose equivalence ratio (furosemide to torsemide) produced similar natriuretic effects, aligning with the observations from the main trial. Notably, while higher torsemide doses led to greater natriuresis, they also resulted in increased neurohormonal activation and mild kidney dysfunction without significant improvements in plasma volume or body weight [90]. These mechanistic insights provide a physiological explanation for the lack of clinical superiority of torsemide observed in the TRANSFORM-HF trial, emphasizing the complex interplay between diuretic effects and compensatory mechanisms in patients with HF. Collectively, the findings from GUIDE-IT, TRIUMPH, and TRANSFORM-HF, along with supporting observational evidence, inform contemporary approaches to biomarker-guided care, individualized maintenance diuretic selection, and optimal transition strategies in acute heart failure, as summarized in Table 4.

**Table 4:** Goal-oriented transition of care: biomarkers and maintenance diuretics

Study Name	Study Design	Intervention	Primary Outcome	Key Findings	Contribution to Evidence-Based Management of Acute HF
Prognostic Role of Serum Chloride Levels in Acute Decompensated Heart Failure [18]	Retrospective cohort study of 1,318 acute HF patients at Cleveland Clinic, validated in a separate 876-patient cohort at University of Pennsylvania.	Assessment of admission serum chloride levels and their association with long-term mortality.	Association between admission serum chloride levels and all-cause mortality.	Lower admission serum chloride was independently associated with increased mortality risk. Prognostic value of serum sodium was diminished compared to chloride.	Highlights the prognostic significance of serum chloride levels in acute HF and suggests it may be a stronger predictor of mortality risk than serum sodium.
Effect of Torsemide vs. Furosemide After Discharge on All-Cause Mortality in Patients Hospitalized With Heart Failure The TRANSFORM-HF Randomized Clinical Trial [88]	Open-label, pragmatic randomized controlled trial of 2859 patients across 60 hospitals in the United States.	Torsemide vs. furosemide for maintenance diuretic therapy post-hospitalization.	All-cause mortality over 12 months.	No significant difference in mortality between torsemide (26.1%) and furosemide (26.2%). No significant difference in hospitalizations.	Challenges previous assumptions about torsemide’s superiority over furosemide for post-discharge diuretic therapy in heart failure patients. Highlights need for individualized diuretic therapy selection.
Mechanistic Differences between Torsemide and Furosemide (TRANSFORM-Mechanism) [90]	Multicenter, prospective, observational mechanistic study of 88 patients randomized to oral furosemide or torsemide.	Patients received either furosemide or torsemide with pharmacokinetic and pharmacodynamic assessments at baseline and 30 days.	Kidney bioavailability, natriuresis, neurohormonal activation, and volume status changes.	Furosemide had higher kidney bioavailability (24.8% vs. 17.1%, *P* < 0.001) and longer duration of natriuresis. A 4:1 dose equivalence resulted in similar natriuresis. Higher torsemide doses led to neurohormonal activation and mild worsening of kidney function without improving volume status.	Refines understanding of loop diuretic pharmacodynamics, suggesting no clinical advantage for torsemide over furosemide when appropriately dosed.
Effect of Natriuretic Peptide–Guided Therapy on Hospitalization or Cardiovascular Mortality in High-Risk Patients With Heart Failure and Reduced Ejection Fraction (GUIDE-IT) [91]	Multicenter, randomized, unblinded trial of 894 high-risk HFrEF patients at 45 sites in the United States and Canada.	Therapy titration guided by a target NT-proBNP level of < 1,000 pg/mL vs. usual care.	Composite of time-to-first HF hospitalization or cardiovascular mortality.	Trial stopped for futility. No difference in the primary outcome between groups (HR: 0.98; 95% CI: 0.79–1.22; *P* = 0.88). No significant differences in medication doses, NT-proBNP levels, or adverse events.	Provides evidence that a strategy of routine NT-proBNP-guided therapy is not superior to high-quality usual care for titrating GDMT in high-risk HFrEF patients. Reinforces that clinical assessment and overcoming practical barriers to titration are paramount.

Key: *GDMT* Guideline-Directed Medical Therapy; *GUIDE-IT *Guiding Evidence Based Therapy Using Biomarker Intensified Treatment in Heart Failure; *HF* Heart Failure; *HFrEF* Heart Failure with Reduced Ejection Fraction; *HR* Hazard Ratio; *NT-proBNP* Aminoterminal pro–B-type Natriuretic Peptide; *TRANSFORM-HF* Torsemide Comparison with Furosemide for Management of Heart Failure; *TRANSFORM-Mechanism* Torsemide Comparison with Furosemide for Management of Patients With Stable Heart Failure

### Opportunities for Improvement and Implementation into Practice

Integrating biomarkers and intensifying GDMT into discharge planning for patients hospitalized with acute HF offers a more objective measure of readiness for outpatient transition, complementing traditional clinical assessments. This approach ensures adequate decongestion and initiation of GDMT before discharge, potentially reducing early readmissions. Utilizing biomarker trends for post-discharge therapy adjustments allows for personalized management, enabling proactive interventions based on biomarker levels even before clinical symptoms manifest. By combining biomarker-guided therapy with individualized diuretic management and comprehensive patient education, healthcare providers can significantly improve the transition of care for HF patients. This integrated approach has the potential to reduce readmission rates, enhance long-term outcomes, and ultimately improve the quality of life for patients with HF.

## Conclusions and Future Outlook

Clinicians often underprioritize complete decongestion — not because it lacks importance, but due to avoidable barriers such as fear of worsening renal function, misinterpretation of clinical signs, diuretic inertia, and system pressures to expedite discharge. Despite a decade of efforts to refine decongestion strategies beyond routine intravenous loop diuretics, we have yet to consistently translate these into improved clinical outcomes, even though successful decongestion is consistently associated with better prognosis. It is critical to recognize that decongestion is not in competition with GDMT, and can improve with better assessments of congestion, kidney dysfunction, and adequacy of diuretic response. Effective decongestion with segmental nephron blockade and intentional GDMT initiation with natriuretic peptide guidance are key components and prerequisites that enable long-term clinical benefits.

## Key References

- Mebazaa A, Davison B, Chioncel O, Cohen-Solal A, Diaz R, Filippatos G, Metra M, Ponikowski P, Sliwa K, Voors AA, et al. Safety, tolerability and efficacy of up-titration of guideline-directed medical therapies for acute heart failure (STRONG-HF): a multinational, open-label, randomised, trial. Lancet. 2022;400:1938-1952. doi: 10.1016/S0140-6736(22)02076-1
  - The STRONG-HF trial demonstrated that rapid, intensive up-titration of guideline-directed medical therapy after acute heart failure hospitalization significantly improved survival and reduced readmissions, setting a new standard for early optimization of foundational therapies. These findings highlight the critical role of early, protocolized GDMT initiation and close follow-up to overcome therapeutic inertia in high-risk patients.
- Mullens W, Dauw J, Martens P, Verbrugge FH, Nijst P, Meekers E, Tartaglia K, Chenot F, Moubayed S, Dierckx R, et al. Acetazolamide in Acute Decompensated Heart Failure with Volume Overload. N Engl J Med. 2022;387:1185-1195. doi: 10.1056/NEJMoa2203094
  - The ADVOR trial showed that adding acetazolamide to intravenous loop diuretics, a segmental nephron blockade strategy, led to faster and more complete decongestion in acute heart failure without increased adverse effects. This evidence supports combining diuretics targeting different nephron segments to enhance decongestion and overcome diuretic resistance.
- Ter Maaten JM, Beldhuis IE, van der Meer P, Krikken JA, Postmus D, Coster JE, Nieuwland W, van Veldhuisen DJ, Voors AA, Damman K. Natriuresis-guided diuretic therapy in acute heart failure: a pragmatic randomized trial. Nat Med. 2023;29:2625-2632. doi: 10.1038/s41591-023-02532-z
  - PUSH-AHF provided the first randomized evidence that natriuresis-guided diuretic adjustment safely improves decongestion in acute heart failure, although without impact on long-term mortality or rehospitalization. These results underscore the value of urine sodium monitoring as an objective tool for personalizing and optimizing diuretic therapy.

## Data Availability

No new datasets were generated or analyzed. All data supporting the findings of this study are available within the cited articles.
